# Resilience of Small Ruminants to Climate Change and Increased Environmental Temperature: A Review

**DOI:** 10.3390/ani10050867

**Published:** 2020-05-17

**Authors:** Aleena Joy, Frank R. Dunshea, Brian J. Leury, Iain J. Clarke, Kristy DiGiacomo, Surinder S. Chauhan

**Affiliations:** Faculty of Veterinary and Agricultural Sciences, The University of Melbourne, Parkville, VIC 3010, Australia; aleenajoyj@student.unimelb.edu.au (A.J.); fdunshea@unimelb.edu.au (F.R.D.); brianjl@unimelb.edu.au (B.J.L.); iain.clarke@unimelb.edu.au (I.J.C.); kristyd@unimelb.edu.au (K.D.)

**Keywords:** adaptation, climate change, heat stress, small ruminants, thermotolerance

## Abstract

**Simple Summary:**

Small ruminants are critical for food security and livelihood, especially under extreme stressful and diverse climatic environments. Generally, sheep and goats are farmed on grazing land in relatively large groups relying on low inputs in terms of feed, water and labor, and possess high thermotolerance compared to large ruminants such as cattle. Climate change has been recognized as a harmful factor influencing sheep and goat production. Small ruminants are vulnerable to direct and indirect effects of climate change, including heat stress, limited and low-quality pasture availability and emerging infectious diseases. In this context, selection of animals for thermotolerance is one viable strategy that exploits natural variation within and between breeds for desirable traits. The various biological markers used to improve thermotolerance in small ruminants include behavioral (feed intake, water intake), physiological (respiration rate, rectal temperature, sweating rate), hormonal (T3, T4 and growth hormone) responses and the response of molecular regulators.

**Abstract:**

Climate change is a major global threat to the sustainability of livestock systems. Climatic factors such as ambient temperature, relative humidity, direct and indirect solar radiation and wind speed influence feed and water availability, fodder quality and disease occurrence, with production being most efficient in optimal environmental conditions. Among these climatic variables, ambient temperature fluctuations have the most impact on livestock production and animal welfare. Continuous exposure of the animals to heat stress compromises growth, milk and meat production and reproduction. The capacity of an animal to mitigate effects of increased environmental temperature, without progressing into stress response, differs within and between species. Comparatively, small ruminants are better adapted to hot environments than large ruminants and have better ability to survive, produce and reproduce in harsh climatic regions. Nevertheless, the physiological and behavioral changes in response to hot environments affect small ruminant production. It has been found that tropical breeds are more adaptive to hot climates than high-producing temperate breeds. The growing body of knowledge on the negative impact of heat stress on small ruminant production and welfare will assist in the development of suitable strategies to mitigate heat stress. Selection of thermotolerant breeds, through identification of genetic traits for adaption to extreme environmental conditions (high temperature, feed scarcity, water scarcity), is a viable strategy to combat climate change and minimize the impact on small ruminant production and welfare. This review highlights such adaption within and among different breeds of small ruminants challenged by heat stress.

## 1. Introduction

Climate change represents a significant global threat to ecosystems and it is predicted that abnormal weather patterns may eliminate 8% of animal species [1]. There are numerous ways that climate change affects grazing livestock particularly through thermal stress, which alters the quality and quantity of pasture and increases the occurrence of pests and disease. Each of these can compromise both livestock productivity and welfare [2,3]. Thus, a detailed examination of the direct and indirect impact of climate change on livestock production, along with an understanding of mechanisms adopted by animals during extreme weather conditions, is of fundamental importance. Comprehensive approaches also allow forecasting and mitigation of the impact of climate change on livestock [4]. Selection of livestock adapted to extreme climatic conditions is one strategy that may sustain livestock production in the future [3].

Livestock production is influenced by a multitude of factors, including physical aspects of an animal’s environment, such as temperature, relative humidity, air velocity, and intensity of solar radiation. Farm animals have a range of thermal comfort zones within which they can produce optimally and this varies with species, breed, age and physiological status [5,6]. The net impact of thermal radiation on animals is influenced by factors such as shade, ground cover and morphological characteristics of the individual [7]. Certain sheep and goat breeds are found to be adapted to hot environments, enabling acceptable rates of productivity [8]. The comparatively small body size of these species, associated low water and feed requirements, good feed conversion ratio, and the capacity to convert low quality feed into quality products are positive features [8].

Optimal nutrition may allow adaption to adverse environmental conditions, ensuring adequate energy supply throughout periods of heat load [9]. Extensively reared livestock is solely dependent on rangeland and developed pasture for their nutritional requirements, so low feed and water availability during summer compromises production, especially in small ruminants of low genetic potential, which are found in arid/semi-arid areas [10]. Therefore, identification of tolerant breeds for higher adaptive capability in extreme environmental conditions (high temperature, feed scarcity, water scarcity) is a viable strategy to mitigate the impact of climate change on small ruminant production. The relative adaptive capacity of different breeds in terms of production and reproduction is a promising area of investigation. Here, we review what is known about production and adaptation in different breeds of sheep and goats under heat stress.

## 2. Scenario of Small Ruminant Production

Approximately 50% of the population of sheep and goats is found in the arid regions of the world, which indicates their versatility and possible tolerance to rising temperatures [11]. These small ruminants produce a wide range of products including meat, milk, fiber, and hide and act as a buffer to environmental fluctuations, reducing financial risks for farmers. Globally, the contribution of milk from goats (3.4%) and sheep (3.4%) is much lower than that of cattle (85%) [12], but 80% of the total goat population is distributed in the tropical areas of Asia and Africa, meeting the dairy demands of low-income communities [13]. In 2013, the global sheep and goat population produced more than 13 million tons of meat and 28 million tons of milk [14]. Furthermore, the production of goat and sheep milk has increased over the last two decades, by 1.3%/annum and 1.7%/annum, respectively [14]. Small ruminants also display relatively good feed conversion efficiency and thermotolerance [15], with goats being adapted best to tropical environments [13].

## 3. Heat Stress as a Major Threat for Small Ruminant Production

High environmental temperatures compromise productivity in lactating sheep and goats, increasing maintenance energy requirements by 7–25%, due in part to higher respiration rate (RR) [16]. Combined effects of lower feed intake and higher energy requirements under thermal stress induce mobilization of stored fat and labile body protein reserves to provide amino acids for protein synthesis and carbon sources for gluconeogenesis [17]. High RR is associated with decreased total protein and cholesterol and increased free fatty acid concentrations, indicating increased protein and lipid catabolism, to meet high energy demands [18,19]. In addition to displaying higher free fatty acid levels, heat stressed animals also increase hepatic glucose output by enhancing glycolysis, glycogenolysis and gluconeogenesis [20]. Both heat stress and the advancement of lactation coincide with declining mobilization of body reserves for milk synthesis, reducing milk yield and quality [21]. In Sarda ewes, for example, a 15% reduction in total milk production was recorded when temperatures were above 21–24 °C and milk production was reduced by 20% if temperature–humidity index (THI) increased from 60–65 to 72–75 [22]. Likewise, Saanen goats exposed to severe heat stress (THI values of 81 and 89) displayed a reduction of 3% and 13% in milk yield, respectively [23]. In Alpine goats in Brazil, heat stress reduced milk yield by 6% [24]. Milk from sheep is primarily utilized for cheese production, which requires relatively high fat and protein content. Thus, it is salient that heat stress reduces both fat and protein content of milk [16]. Significantly lower milk fat and protein yields were also found in temperate Alpine and Nubian goats during the summer season [25].

Warm environments are believed to compromise udder health and milk quality in ewes and it is very likely that microorganisms involved in the clinical or sub-clinical mastitis or contaminations of ewe udder are the cause [16]. Furthermore, exposure to intense solar radiation adversely affects the hygienic quality of milk. Increased bacterial load in milk is represented by higher quantities of pathogenic microorganisms and milk neutrophils, increasing the somatic cell count. Hence, heat stress can compromise mammary defense capacity, leading to greater bacterial colonization in ewe udders, and lower milk quality [16].

Heat stress may also affect meat productivity and organoleptic quality of meat from both sheep and goats. Activation of the adrenergic system, initiated by heat stress, leads to peripheral vasodilation and muscle glycogenolysis, both of which result in higher pH and darker meat in heat-stressed ruminants [26]. The higher ultimate pH and shear force of meat from heat stressed goats increased cooking loss, reduced water holding capacity, and increased toughness [27]. Reduced weight of edible (liver, blood, heart, intestine) and inedible (skin, feet) components have also been noted in heat-stressed sheep and goats [27,28].

The production and reproductive loss in small ruminants during heat exposure may be partly explained by lower feed intake but is also influenced by altered endocrine and metabolic status [29]. Elevated body temperature in rams during heat stress causes testicular degeneration, reduction in percentage of normal and fertile spermatozoa, low ejaculate volume, high semen pH, diminished sperm motility, increased incidence of abnormal sperm and reduction in sperm quality [30,31]. The reproductive function of rams is compromised above ambient temperatures of 40 °C for 1.5–2 h [29]. Heat stress also reduces the intensity of sexual behavior in ewes and may lead to failure of the animal to conceive and maintain pregnancy [32]. Hot environments, coupled with compromised nutrition may result in estrous cycles of reduced length, increased production of abnormal ova, lower conception rates and early embryo mortality in ewes [29]. In summary, heat stress limits a variety of production traits and reproduction in small ruminants and timely interventions are necessary to improve welfare and production during summer months.

## 4. Thermoregulatory Mechanisms in Small Ruminants

Like other homeothermic animals, sheep and goats maintain their core body temperature within a narrow range best suited to carry out normal body functions. In order to sustain optimal production, animals should maintain relatively constant body temperature under various environmental conditions with minimum additional energy expenditure. Small ruminants demonstrate a wide range of adaptive responses to overcome the direct and indirect impact of heat stress in tropical regions. Often high ambient temperature during summer initiates intense thermoregulatory responses, allowing dissipation of extra heat. Some of the commonly seen responses in small ruminants include modified behavior and physiological/biochemical processes to counteract the adverse effects of heat and thereby maintaining thermal equilibrium [33].

Various adaptive responses, including reduced feed intake, rumination time and frequency, and increased water intake, enable small ruminants to cope with adverse environmental conditions [34,35]. Such responses are invoked to reduce internal heat production due to metabolism. Physiological means of coping with elevated environmental temperatures include enhanced RR, sweating rate (SR), and pulse rate (PR) [36,37]. The biochemical profile of ruminants is also modified under hot environments, with changes in hematocrit values, hemoglobin and lowered blood levels of glucose, protein, cholesterol and non-esterified fatty acids (NEFA) [38].

At the molecular level, adaptive responses to heat exposure are driven by increased expression of heat shock proteins (HSPs), which function as intra-cellular chaperones and prevent protein and cell damage in heat-stressed animals. Several studies have reported higher expression of *HSP 70*, *HSP 60, HSP 90* and *HSP 27* genes in small ruminants during heat stress [33,39,40]. Similarly, Mohanarao et al. [40] also showed higher expression of *HSPA1A, HSPA1L, HSPA6* and *HSPA8* genes in goats with heat stress. Various experiments conducted in small ruminants have also demonstrated compromised or enhanced immune cell activity during summer [41]. Thus, lower expression of the cytokine genes interleukin 18 (*IL-18*), tumor necrosis factor-*α* (*TNF-α*), interferon-*β* and interferon-*γ* has been reported [41,42]. Expression of intracellular toll-like receptors (TLRs) is also reduced in heat-stressed goats [43,44]. Savitha et al. [45] suggested a higher expression of *TLR3, TLR3, TLR7, TLR8* and *TLR9* as reliable immunological markers for the indication of the severity of heat stress in goats. Higher expression of interleukin type 1 receptors (*IL1R1, IL1R2*) and heat shock-related 70-kDa protein 2 (*HSPA2*) are also seen in heat-stressed sheep, suggesting that these genes also play an important role in the immunological responses [46].

Hormones, especially those produced from the pituitary (prolactin), adrenal (cortisol) and thyroid glands (Triiodothyronine—T3 and Thyroxine—T4), play a major role in thermoregulation and metabolic adjustments in animals, in hot environments. The activation of the hypothalamo-pituitary-adrenal axis (HPA axis) may lead to enhanced production of corticotropin-releasing hormone (CRH) in the hypothalamus, which stimulates adrenocorticotropic hormone (ACTH) from the anterior pituitary, in turn causing increased cortisol secretion from the adrenals [47]. Antidiuretic hormone (ADH) expression is also increased, reducing excretion of water [47]. Cortisol is the primary stress hormone in ruminants [38] and Sheep and goat breeds that are indigenous to the semi-arid tropical regions display higher thermotolerance in temperatures of 40 °C and 42 °C by increasing cortisol production [10,48]. An increase in cortisol levels in response to heat stress causes protein catabolism to provide glucogenic amino acids utilized in gluconeogenesis [49]. This is, however, not a reliable indicator of heat stress, since not all studies in small ruminants show elevation of plasma levels of cortisol. For example, we [42] studied the stress response in Australian Merino cross-bred wethers exposed to temperatures of 40 °C for one week and did not observe an elevation in cortisol levels. On the other hand, Wojtas et al. [50] studied Polish Merinos exposed to temperatures of 40 °C for two weeks and observed elevated levels of cortisol in blood during the first but not second week of heat stress. Likewise, in Holstein heifers, heat stress did not cause elevation, actually causing a reduction in cortisol levels [51].

Prolactin is another multifunctional hormone involved in a variety of biological functions related to thermoregulation in small ruminants. Higher circulating prolactin concentrations in ruminants under hot conditions may indicate the possible modulatory role of prolactin for thermal acclimatization and prolactin is also identified as a reliable marker of heat stress [52]. Horrobin [53] reported on the critical role of prolactin for water conservation as it helps to diminish renal fluid and electrolyte excretion under hot environments. In addition, Choy et al. [54] demonstrated a significant association between HS and increased prolactin mRNA expression in ovine sweat glands, further suggesting that the prolactin response is important for heat dissipation by enhancing sweat gland activity. However, prolactin’s role as a thermoregulator in heat-stressed ruminants requires elucidation. Notably, elevated prolactin levels are a universal feature of heat stress in goats [55], cows [51,56], sheep [57,58] and humans [59,60] indicating that this parameter may be used as a reliable measure of heat stress.

Depressed thyroid activity is another feature of heat stress in small ruminants [61,62], accounting for lower metabolic activity, reduced rumen motility and decreased rate of passage. Peripheral thyroid hormone (T3 and T4) concentrations are lower in ruminants during summer than in winter [62], which could be an adaptive mechanism to reduce metabolic heat production during warmer months [61]. Additionally, leptin, adiponectin, growth hormone, mineralocorticoids and ADH are involved in thermal adaptation in small ruminants and could also act as indicators of thermal stress [38].

Recently, there has been an increasing interest in animal diet supplementation with different feed additives such as antioxidants, electrolytes, vitamins and betaine for ameliorating the impact of heat load in small ruminants [37,63,64]. Reformulation of diet can greatly help to improve welfare of an animal by modifying thermoregulatory responses [65]. Studies conducted in our lab have shown that supplementing heat stressed sheep with betaine improved thermoregulatory responses by maintaining lower RR, rectal temperature (RT), skin temperature (ST) and heart rate (HR) compared to animals fed with normal ration [37]. Furthermore, vitamin E [55], C [66] and a combination of vitamin E and Selenium [64] supplementation to small ruminants could reduce heat stress symptoms and improve animal welfare. Goats fed with nutrient-dense diets (supplemented with 4% fat and Soybean oil) showed lower RT and higher milk fat content during heat stress treatments [67]. A few studies also reported that ewes supplemented with high levels of saturated and monounsaturated fatty acids or algae-derived docosahexaenoic acid during late gestation reduces newborn lamb thermogenesis [68,69]. On balance, the literature indicates that advances in feeding strategies could ameliorate metabolic and physiological disturbances in small ruminants during high heat load.

## 5. Genetic Differences in Heat Tolerance in Small Ruminants

Sheep and goats are more able to tolerate severe heat stress and periods of water and feed scarcity, where other species struggle to survive [70]. There are more than 1000 sheep and 600 goat breeds throughout the world and the capacity to adapt to the thermal challenge differs between the breeds. Tropical sheep breeds are more highly adapted to arid and semi-arid regions, having efficient thermoregulatory mechanisms [15]. Sheep and goat breeds that tolerate extreme heat and dehydration can walk longer distances in search of food and are able to survive on limited pastures during summer [15]. Therefore, the selection of breeds for thermotolerance is a valuable means of sustaining small ruminant production with a changing climatic scenario.

The adaptive capacity of animals is generally evaluated by assessing the ability to produce under extreme climates, and the capacity to show minimal deleterious effects on production, lower mortality rates and maintenance of reproductive efficiency [71]. Indigenous breeds that have evolved in tropical and subtropical regions have a higher adaptive capacity to such stress than the exotic breeds. The higher feed conversion ability on low-quality pastures of Damara sheep, compared to Merinos, is such an example [72]. Fat-tailed Damara sheep fed low-quality feed showed greater (10%) dry matter digestibility and energy conversion [72], indicating the better ability of this breed to meet the nutritional challenges under the expected adverse climatic conditions in future.

Relatively higher production performances of the heat-stress adapted goat breeds were revealed in comparative studies between Alpine and Nubian goat breeds. In similar environmental conditions (34 °C temperature and 25% humidity; THI = 79), the milk yield of Nubian goats, indigenous to tropical regions, was maintained, while that of Alpine breeds was reduced as temperature elevated from 27 °C to 34 °C [25]. Furthermore, Lallo et al. [73] recorded better production efficiency of Anglo-Nubian goats compared to Alpine goats in hot conditions, highlighting the potential of crossbreeding programs to minimize production losses due to climate change effects. In addition, Hamzaoui et al. [35] showed steady milk yield in heat stressed Murciano–Granadina dairy goats compared to goats under thermoneutral conditions. Although the milk production in tropical breeds is less than that of exotic temperate breeds, the rate of reduction in milk yield and quality of milk produced is negligible in these breeds during summer [8].

In addition to milk yield, the growth rate of the animals is an important production parameter as it directly represents the overall quantity of meat produced. A comparative assessment study between Baladi and Damascus goat breeds showed a greater rate of reduction in body weight in the latter (2.85% vs. 3.33%) under thermal challenge [74]. Pragna et al. [75] also reported a reduction in body weight and body condition score among indigenous goat breeds under heat challenge, with the effect being greater in the Osmanabadi breed than Malabari and Salem Black breeds. Malabari and Salem Black breeds appear to tolerate arid conditions whilst sustaining productivity. Furthermore, superior thermotolerant capacity of the native Indian semi-arid sheep breed (Malpura) to heat stress challenges has also been demonstrated such that the Malpura displays no compromise of growth when challenged with heat stress compared to sheep maintained at thermoneutral conditions [15,36]. In a comparative study between Dorper and second cross Merino lambs (Poll Dorset × Merino/Border Leicester), we [76] have observed some breed differences for meat quality in response to heat stress. Dorpers exposed to heat stress showed lower water holding capacity than the thermoneutral group, while this effect was not observed in second cross lambs. Furthermore, heat stress increased muscle redness in second-cross Merinos compared to thermoneutral lambs during 10 d retail display period.

Generally, differences between breeds in adaptation to heat stress can be attributed to 3 main factors: adaptation to elevated temperature and radiation through efficient behavioral and physiological thermoregulation, adaptation to water restriction, with capacity to withstand severe dehydration, and adaptation to periods of feed scarcity, supported by small body size, low and variable metabolic requirements, efficient digestive capacity and skillful grazing behavior [77].

According to Gandhi and Arjava [78], tropical breeds are comfortable in environmental temperatures as high as 38 °C, while temperate breeds perform better within a temperature range of 5–25 °C. Tropical breeds possess higher thermo-tolerance by virtue of particular thermoregulatory functions such as low metabolic heat production and high heat dissipation [73,77]. For example, Malpura ewes evolved in the Indian semi-arid regions showed extreme adaptability to heat stress, nutritional stress and combined stress (heat stress + nutritional stress) conditions (temperature—40 °C; humidity—55%) by adjusting their RR [10,15]. A similar display of lower physiological responses was also reported in Santa Inês and Morada Nova sheep breeds during summer in semi-arid environments of Brazil [79]. Sheep breeds adapted to desert regions (bighorn desert sheep) compensate for higher water loss in heat stress conditions through the production of highly concentrated urine [80]. In addition, dehydration during summer may lead to slower digestion of feed in the digestive tract, ultimately conserve body water loss in drought-adapted sheep breeds [34]. Genome wide analysis of candidate genes for desert environment adaptation in 77 Chinese native sheep breeds documented various genetic pathways that are functionally important for drought tolerance [81]. The functions of the candidate genes were classified into four pathways, being the arachidonic acid metabolism pathway (*ANXA6*, *GPX3, GPX7* and *PTGS2*), the renin–angiotensin system pathway (*CPA3, CPVL* and *ECE1*) and the oxytocin signaling pathway (*CALM2*, *CACNA2D1*, *KCNJ5* and *COX2*), having a specific roles in the conservation of water under drought challenges [81]. However, breeds that evolved in a similar region also show differences in their adaptive capacity to stressful conditions. For example, Awassi sheep are more drought tolerant than Najdi sheep (domestic sheep breeds primarily reared in Saudi Arabia) [52]. Barbados Blackbelly sheep, which originated from West Africa, are well known for resilience while maintaining acceptable production characteristics in the extreme tropic environments [82]. Interestingly, these adaptive traits are also noteworthy when used for crossbreeding and are the dominant breed in the Caribbean islands, also having been exported to other countries like Mexico, Venezuela, the USA and Indonesia [82,83]. They can graze even at hot temperature and humidity, are able to survive on poor quality tropical grasses and possess better tolerance to gastrointestinal parasite infections that would severely affect other breeds even evolved from tropical regions [82,84]. Likewise, St. Croix sheep, native to the US, are adapted to a wide range of climatic environments ranging from hot humid climate of tropics (shedding hair during summer, which helps in efficient thermoregulation) and can also survive in cold temperatures (growing a thick wool coat in winter) [85]. Therefore, selection for the genetic traits for higher adaptive capability in extreme environmental conditions could be a promising strategy to mitigate the impact of climate change on small ruminant production.

### 5.1. Morphological Adaptation

Morphological traits in livestock are crucial from the adaptation point of view as they directly influence the heat exchange mechanisms (cutaneous convection, radiation, and evaporation) between the animal and surrounding environment [71]. Morphological adaptive characteristics vary between breeds evolving in divergent environmental regions [71]. Coat color is considered as a qualitative index indicating the genetic superiority of animals to hot environments [80]. Animals with light/white colored coats have an advantage in tropical regions, because the coat reflects 50–60% of direct solar radiation, in contrast to dark-colored animals [71]. Barbari goat breeds have a white coat color and small body size and show better thermotolerance than other goat breeds such as the Sirohi and Jhakrana reared and developed under similar regions [86]. Fadare et al. [87] demonstrated the lower thermotolerance of darkly pigmented sheep breeds. Darkly pigmented breeds showed higher RT, RR, PR and red blood cell count compared to lightly pigmented sheep breeds. Coat length, thickness and hair density may also affect the adaptive ability of animals in tropical regions. Short hair, thin skin and a lower number of hairs per unit area are directly linked to higher adaptability to hot conditions [88]. Indigenous sheep breeds adapted to arid and semi-arid regions possess morphological characteristics such as carpet type wool (Marwari, Omani and Barbarine), which seems to provide better protection from direct solar radiation, as well as allowing effective cutaneous evaporative heat dissipation [88,89].

Morphological traits including low coat thickness, absence of wool, large sweat glands and a smaller number of hairs in hair breeds of sheep make them more thermo-tolerant than the wool-bearing counterparts [71]. The lower metabolic heat production and slower and deeper breathing pattern in hair sheep breeds under heat stress also allows adaption to hot environments [62]. Fat tail sheep are recognized as being morphologically adapted to hot environments, as the external localization of the body fat allows better heat transfer by other body parts [90]. Additionally, fat stored in the tail also acts as an energy and water source in these breeds during periods of dietary restriction. A comparative study between three Indian goat breeds showed differences in their rump height, which is higher in the Salem Black (more tolerant) breed than the Malabari and Osmanabadi breeds [75]. Chacón et al. [91] suggested that this as an evolutionary adaptation which assists in avoidance of reflected heat radiation from the ground, while grazing under hot conditions.

### 5.2. Behavioral Adaptation

A comparative study among tropical indigenous goat breeds (Osmanabadi, Malabari, and Salem black) showed differences in feed intake during the summer season, being reduced in Malabari animals but not the other breeds during heat stress [75]. Likewise, heat stress reduced feed intake in second-cross Merino (Poll Dorset × Merino/Border Leicester) lambs, but not Dorper lambs when both breeds were subjected to heat stress [92]. The authors attributed lower feed intake to increased severity of heat stress experienced by the susceptible breed as this could possibly be an effort to reduce metabolic heat production. Alamer and Al-hozab [52] established a difference in drought tolerance ability between the Awassi and Najdi sheep (local breeds of Saudi Arabia), with better water conservation in the former.

### 5.3. Physiological Adaptation

Generally, it is reported that genetic adaptation of thermotolerant breeds allows them to maintain lower physiological limits compared to those being more susceptible to heat stress under similar environmental conditions. Factors such as RR, RT and HR are identified as critical physiological indices to identify levels of thermotolerance in small ruminants [10,36]. Breed differences have been suggested in sheep based on physiological adaptation. In our recent comparative study, Dorper lambs had lower RR, RT and ST than second cross Merinos (Poll Dorset × Merino/Border Leicester) lambs exposed to heat stress treatment [92]. Likewise, according to Srikandakumar et al. [93], Omani sheep display lower RT and RR than Merinos under both cold and hot environments. The lower RR indicates that both the Dorper and Omani breeds are more economical in terms of diverting energy to the productive traits rather than for adaptation. Tropical sheep breeds reared in the semi-arid regions of India are able to maintain lower RT (38.9 °C vs. 39.4 °C), RR (40 vs. 108 breaths/min) and PR (82 vs. 96 beats/min) than Merinos under thermal stress (33.7 °C; 54.9% vs. 30.1 °C; 48.6%) [50,94]. Similar variations in RT, RR and PR have been reported for different goat breeds [33,95]. Thus, lower RT and RR even under thermo-neutral conditions identify thermotolerant breeds. In addition, Alamer and Al-hozab [52] reported that SR is lower in Awassi sheep than in Najdi sheep under summer conditions and this can be used as a selection criterion. The reduced SR in Awassi sheep compared to Najdi breed indicates better water conservation ability and drought tolerance. Rout et al. [95] recommended the identification of contrasting phenotypes within a breed to remove wide individual variation among animals in response to environmental stimuli. The broad range of variability for both RR (24–108) and PR (80–160) in response to hot conditions enable the use of these variables as reliable stress indicators to detect contrasting phenotypes within a breed of small ruminants [95].

### 5.4. Cellular and Molecular Adaptation

Thermal acclimatization and adaptation in small ruminants are always associated with higher HSP levels, which could be considered as a cellular thermometer [95]. Cells are better adapted to thermal stress if HSP concentrations are high and are increasingly vulnerable when HSP levels are low. However, it is not yet proven that breeds better adapted to stressful conditions have lower or higher *HSP* synthesis than more susceptible breeds. A few studies have demonstrated higher *HSP 70* expression in heat-tolerant breeds. For example, when Suffolk and Pelibuey sheep breeds were compared for thermotolerance ability, the latter had higher *HSP 70* expression and more efficient thermoregulation than the former [96]. In addition, higher cell survival rates are seen in Pelibuey than in Suffolk sheep, which could be related to their effective *HSP 70* adaptive response. Nevertheless, Aleena et al. [33] documented lower *HSP 70* mRNA expression in adapted Salem Black goats as compared to Malabari and Osmanabadi breeds. Similarly, Kumar et al. [86] observed lower expression of *HSP 70*, *HSP 60* and *HSP 90* in heat adapted Barbari goats than in less adapted Sirohi and Jhakarana breeds. Relatively higher *HSP* gene expression level in susceptible breeds probably indicates a greater requirement to protect cells from heat stress damage.

In Osmanabadi, Salem Black and Malabari goat breeds, insulin-like growth factor 1 (*IGF-1*) mRNA expression were reduced under thermal stress, with the rate of reduction being lower in the Salem Black breed [75]. This could indicate better adaptability of Salem Black breed over the other two breeds in terms of sustaining productivity in hot environments. Heat stress has also been reported to compromise the innate immune functions in small ruminants demonstrated by down-regulation of TLRs, *interleukin 1β*, *TNF-α*, *interferon-β* and *interferon-γ* [41,43]. In terms of immune competence, Savitha et al. [45] recorded higher expression of *TLR3*, *TLR7*, *TLR8* and *TLR9* in Salem Black goats than in Osmanabadi goats indicating superior thermotolerance of former as it was still able to express a better immune response (indicated by greater expression of TLRs) as compared to Osmanabadi goats during heat stress conditions.

### 5.5. Endocrine Adaptation

Plasma cortisol concentrations vary considerably between various goat breeds during the summer season. Archana et al. [27] recorded increased plasma cortisol concentration in the Osmanabadi goats during summer, while no effect of heat stress was observed in Salem Black goats. The lower plasma cortisol concentrations in Salem Black goats indicate their superior adaptability to stressful conditions. Breed variations are also evident for plasma T3/T4 concentrations during summer. Crossbred-Chokla sheep had higher plasma T3 concentrations during summer than the respective pure breeds [97], which could be indicative of aberrant thyroid gland activity and poor thermoregulation of exotic breeds in hot environments. In a comparative study of Barbados Blackbelly (tropical breed), Dorset (temperate breed) and Blackbelly × Dorset cross, lower plasma T4 concentrations were seen in Barbados Blackbelly and Blackbelly × Dorset crosses under heat stress (compared to levels in thermoneutral conditions), while the rate of change was higher in the Dorsets [98]. This led the authors to conclude that those breeds which reduce plasma thyroid hormone concentrations during heat stress are more adaptable to hot environments, due to a reduction in metabolic heat production. Heat stress significantly increased plasma growth hormone concentrations in different goat breeds (Osmanabadi, Salem Black and Malabari) with the greatest concentrations in Osmanabadi breed than both Salem Black and Malabari goats [75]. Higher plasma growth hormone concentrations in Osmanabadi breeds could be related to their increased thermotolerance compared to other breeds and were able to achieve higher growth rate and performance.

### 5.6. Metabolic Adaptation

The small size of the tropical breeds probably reflects natural selection for genotypes that adapt to the available feed resources in the tropical arid regions [77]. The lesser requirement for nutrients enables survival when feed is of low quality and quantity during summer in arid and semi-arid areas [10]. The relatively small size also ensures limited metabolic requirements, with lower heat production related to metabolism. This adaptation of tropical breeds confers a local advantage. Volatile fatty acid profiles in the rumen are important determinants of energy supply in ruminants. A comparative study demonstrated higher propionate production in Salem Black animals than in both Osmabnabadi and Malabari breeds [61], which would cause less methane synthesis. Differences in the proportion of volatile fatty acids concentration also demonstrate the breed variations in the diet digestibility and rumen microbial population in goats exposed to heat stress challenges [61].

### 5.7. Blood Biochemistry and Adaptation

Heat stress reduces blood pCO_2_ in both Omani and Merino sheep, but the effect is greater in Omani. Similarly, blood oxygen saturation was found to be increased in Omani breeds while reduced in Merino. The decreased blood oxygen concentrations in Merino breeds could be due to their fast-shallow respiration pattern as these breeds were not able to saturate more oxygen into the blood by removing CO_2_ because of increased RR [93]. Heat stress also reduced total blood hemoglobin concentrations in Merinos but not Omani; this could be due in part to higher water intake, resulting in hemodilution. In addition, lower blood hemoglobin concentration could also be attributed to low nutrient status in Omani sheep for hemoglobin synthesis resulting from reduced feed intake [93]. Generally, higher activity of Creatinine kinase (CK) in blood plasma indicates muscle damage and fatigue in small ruminants. Among different sheep breeds, Dormer (a breed developed from Dorset Horn and Germany Merino) showed the highest CK activity as compared to Blackhead Persian, South African Mutton Merino and Dorper [99]. The elevated CK activity in Dormers indicates susceptibility to heat stress. Breed differences in thermotolerance in small ruminants, as indicated by changes in various biological markers from adaptation to hot environments, are detailed in Table 1.

## 6. The Potential for Genetic Improvement to Improve Resilience in Small Ruminants

Sheep and goats are selected for meat, fleece and milk production, and breeding objectives also incorporate other functional attributes such as reproductive efficiency and disease resistance. Unfortunately, artificial selection has led to some undesirable results, such as lower thermotolerance and poorer disease resistance. However, higher sensitivity to sudden environmental disturbances has become a major issue due to climate change. Resilience is the capacity of an animal to be minimally influenced by disturbances or to quickly return to the resting state pertaining prior to the disturbance. While small ruminants (sheep and goats) are more thermotolerant than cattle, they remain vulnerable to the direct and indirect effects of climate change. In this context, selection of animals is a cost-effective, cumulative, and permanent strategy that exploits natural variation among breeds for desirable traits. Thus, improved animal genetics can contribute to both adaptation and mitigation approaches to sustain animal production in the changing climate scenario.

Selective breeding has already improved the performance of domesticated ruminants subjected to environmental challenges. The main objective of future selection to sustain small ruminant production should focus on several specific characteristics such as high productivity, improved thermotolerance and high disease resistance. Essential traits that can be considered in such selection could be skin and hair type, sweat gland capacity, reproductive rate, feed conversion efficiency to maintain production in limited pastures, disease and drought tolerance, metabolic heat production and other morphologic and genetic features that can contribute to sustainable small ruminant production [100]. Selection of thermotolerance in small ruminants based on differences in their physiological variables (RT, RR, SR), plasma or serum hormone concentrations (prolactin, cortisol, T3, T4, growth hormone) and genetic markers (HSPs, *IGF-1*, TLRs and ILs), with the addition of THI index in genetic evaluation models, are also promising. However, the selection for adaptive traits indicates that tropical breeds have higher disease resistance, may thrive on low-quality feed and are able to endure heat and drought to a greater extent than temperate breeds [33,75]. Genes having an important role in pathways related to metabolism, immunity, and heat responses could act as potential biomarkers for genetic selection in small ruminants. Further studies of rumen kinetics (volume, particle flow, site of digestion) and the thyroxine status among breeds are needed to identify a potential breed that can withstand the nutritional deficiency during summer. Nevertheless, traditional crossbreeding to introduce thermotolerant genotypes from adapted breeds into high producing breeds would be limited due to the co-introduction of unfavorable traits, particularly those associated with low productivity. Therefore, future selection should aim for a balance between heat adaptation, health and production. Henry et al. [101] suggested an alternative approach to incorporate specific thermotolerant genes into high performing breeds while avoiding undesirable genotypic traits. Incorporation of specific genes to improve thermotolerance requires identifying/mapping of individual genes responsible for better adaptive traits and this approach opens the way towards an improved breeding program through marker-assisted selection and transgenics. Nevertheless, as we reviewed previously [102], the investments and commitment needed to achieve this goal is a shared responsibility, requiring action by stakeholders across all sectors of society.

## 7. Conclusions

Climate change poses a potential threat to small ruminant production. Although sheep and goats can adapt to the stress by enabling utilizing adaptive responses for survival, production is often compromised. Accordingly, appropriate adaptation and mitigation strategies based on recent research potentially offer many opportunities to sustain small ruminant production. Research to date indicates that signatures of natural selection among small ruminant breeds adapted to harsh environmental conditions (high temperature, feed and water scarcity), could be identified and information utilized for genetic improvement in thermotolerance. At a phenotypic and genotypic level, sheep and goat breeds that have evolved under different agro-ecological conditions express various adaptive features, including coat characteristics (color, hair length and density), pigmented skin, fat tail shape, heat and drought tolerance. Future approaches involving the comparison of different breeds using high density genotyping may elucidate underlying biological mechanisms and genetic traits that govern these adaptive characteristics. The integration of these approaches into future small ruminant breeding programs will help in coping with the adverse effects of changing climate. In addition, the effects of climate change are projected to be varied in different locations. Therefore, region-specific investigations should be undertaken with regard to the adaptation of small ruminants in the local stressed climates. The adapted local breeds can be better alternatives as an appropriate bio-resource to sustain small ruminant production under changing climatic conditions.

## Figures and Tables

**Table 1:** Biological markers for breed differences in small ruminants for thermotolerance capacity.

Variable	Species	Tolerant Breed	Susceptible Breed	Quantity of Stress	Effect on Tolerant Breed as Compared to the Susceptible Breed	Reference
**Behavioral Adaptation**						
Water intake	Sheep	Awassi	Najdi	Summer heat stress Temperature—35.3 °C RH—16%	Lower water intake	[52]
	Sheep	Dorper	Second cross Merinos	Cyclic heat stress Temperature—36–40 °C	Lower water intake	[92]
	Goat	Salem Black	Osmanabadi	Summer heat stress THI—86.5	Lower water intake	[33]
Feed intake	Sheep	Dorper	Second cross Merinos	Cyclic heat stress Temperature—36–40 °C	No change in feed intake	[92]
	Goat	Salem Black Osmanabadi	Malabari	Summer heat stress THI—86.5	No change in feed intake	[75]
**Physiological Adaptation**						
Rectal Temperature	Sheep	Omani	Merino	Summer heat stress THI—93 ± 3.1	Lower rectal temperature	[93]
	Sheep	Dorper	Second cross Merinos	Cyclic heat stress Temperature—36–40 °C	Lower rectal temperature	[92]
	Goat	Salem Black	Malabari Osmanabadi	Summer heat stress THI—86.5	Lower rectal temperature	[33]
	Goat	Jamunapari	Barbari	Summer heat stress Temperature—47.5 °C RH—21.5%	Lower rectal temperature	[95]
Respiration rate	Sheep	Omani	Merino	Summer heat stress THI—93	Lower respiration rate	[93]
	Sheep	Dorper	Second cross Merinos	Cyclic heat stress Temperature—36–40 °C	Lower respiration rate	[92]
	Goat	Jamunapari	Barbari	Summer heat stress Temperature—47.5 °C RH—21.5%	Lower respiration rate	[95]
	Goat	Salem Black	Malabari Osmanabadi	Summer heat stress THI—86.5	Lower respiration rate	[33]
Sweating rate	Sheep	Awassi	Najdi	Summer heat stress Temperature—35.3 °C RH—16%	Lower sweating rate	[52]
**Endocrine Adaptation**						
T3	Sheep	Chokla	Cross-breds of Chokla	Summer heat stress Temperature—38.8 °C RH—41.3% THI—78.9	Lower concentration	[97]
T4	Sheep	Barbados Blackbelly	Dorset Blackbelly × Dorset crosses	Temperature—33.8 °C	Lower rate of change in different seasons	[98]
Growth hormone	Goats	Salem Black Malabari	Osmanabadi	Summer heat stress THI—86.5	Higher concentration	[75]
**Cellular and Molecular Adaptation**						
*Leptin gene*	Goat	Jamunapari	Barbari	Summer heat stress Temperature—47.5 °C RH—21.5%	Lower expression	[95]
*HSP 70*	Goat	Salem Black	Malabari Osmanabadi	Summer heat stress THI—86.5	Lower expression	[33]
	Goat	Barbari	Sirohi Jhakarana	Summer heat stress THI—81.63	Lower expression	[86]
	Sheep	Pelibuey	Suffolk	Temperature— 43 °C	Lower expression	[96]
*HSP 60*	Goat	Barbari	Sirohi Jhakarana	Summer heat stress THI—81.63	Lower expression	[86]
*HSP 90*	Goat	Jamunapari	Barbari	Summer heat stress Temperature—47.5 °C RH—21.5%	Higher expression	[95]
	Goat	Barbari	Sirohi Jhakarana	Summer heat stress THI—81.63	Lower expression	[86]
*IGF-1*	Goat	Salem Black	Osmanabadi Malabari	Summer heat stress THI—86.5	Higher expression	[75]
TLR genes (*TLR3*, *TLR7*, *TLR8* and *TLR9*)	Goat	Salem Black	Osmanabadi	Summer heat stress THI—86.5	Higher expression	[45]
